# Integrated network pharmacology and experimental verification to investigate the mechanisms of YYFZBJS against colorectal cancer *via* CDK1/PI3K/Akt signaling

**DOI:** 10.3389/fonc.2022.961653

**Published:** 2022-11-15

**Authors:** Jinxiao Li, Fangyuan Zhou, Luorui Shang, Na Liu, Yuhan Liu, Mengqi Zhang, Shuhan Wang, Shenglan Yang

**Affiliations:** ^1^ Department of Integrated Traditional Chinese and Western Medicine, Union Hospital, Tongji Medical College, Huazhong University of Science and Technology, Wuhan, Hubei, China; ^2^ Rehabilitation Department of traditional Chinese Medicine, Union Red Cross Hospital, Wuhan, China; ^3^ Department of Clinical Nutrition, Union Hospital, Tongji Medical College, Huazhong University of Science and Technology, Wuhan, Hubei, China

**Keywords:** YYFZBJS, colorectal cancer, network pharmacology, PI3K/Akt pathway, apoptosis, cell cycle

## Abstract

**Background:**

Colorectal cancer (CRC) is a common digestive tract malignancy with rising incidence and morbidity worldwide during recent years. Yi-Yi-Fu-Zi-Bai-Jiang-San (YYFZBJS), a traditional Chinese medicine formula, has showed positive effects against cancers. However, the mechanisms underlying its anticancer effects requires investigation.

**Methods:**

Information on bioactive compounds, potential YYFZBJS targets, and CRC-associated genes, was obtained from public databases. The key targets and ingredients as well their corresponding signaling pathways were identified using bioinformatic approaches, including Kyoto encyclopedia of genes and genomes (KEGG) analyses, gene ontology (GO), and protein–protein interaction (PPI). Subsequently, molecular docking was used to verify the main compounds-targets. Potential YYFZBJS therapeutic effects against CRC were validated *in vitro* and *in vivo*.

**Results:**

Using pharmacological network analysis, 40 YYFZBJS active compounds and 21 potential anti-CRC targets were identified. YYFZBJS was an important regulator of CRC through various targets and signaling pathways, particularly the cell cycle and PI3K/AKT pathway. Additionally, YYFZBJS suppressed the proliferation of CRC cells. Flow cytometry showed that YYFZBJS induced apoptosis and cell cycle arrest in the G2/M phase. Western blotting analysis indicated that YYFZBJS reduced the protein levels of CDK1, p-AKT, and p-PI3K, without altering total PI3K and AKT protein levels. *In vivo* analysis found that YYFZBJS inhibited tumor growth and PI3K/AKT signaling in a mouse model of CRC.

**Conclusion:**

As predicted by network pharmacology and validated by the experimental results, YYFZBJS inhibited proliferation, induced apoptosis and arrested cell cycle progression in CRC by modulating the CDK1/PI3K/Akt signaling pathway.

## Introduction

Ranked as the 3^rd^ leading malignant tumor globally, colorectal cancer (CRC) is considered a fatal disease with mortality rates. Among the symptoms of CRC patients, the most common are abdominal pain, anemia, and rectal bleeding (1). CRC accounts for about 10% of annual cancer diagnoses and deaths (2). The rise of CRC incidence is attributed to risk factors like ageing populations, obesity, dietary habits, and limited physical activity (1). CRC is treated using surgery, chemotherapy, radiotherapy, and immunotherapy. However, these strategies are associated with disease recurrence, metastasis, and severe side effects. Thus, more effective treatments, without severe adverse effects should be developed.

Traditional Chinese medicine (TCM) is used as an adjunctive therapeutic option for preventing CRC metastasis, enhancing chemotherapeutic effects while minimizing toxicity, and increasing the patients’ quality of life (3). Furthermore, due to its effectiveness and few side effects, the use of TCM for oncological applications has been rising in Western countries. Yi-Yi-Fu-Zi-Bai-Jiang-San (YYFZBJS), a classical TCM formula, was first described in Golden Chamber which was written by a TCM master, Zhang Zhong-Jing (Eastern Han Dynasty, A.D. 150–219). It’s made up of three traditional Chinese medicines, Yi-yi-ren (Semen Coicis), Fu-Zi (monkshood), and Bai-jiang-cao (Herba Patriniae), which are combined at a 30:6:15 ratio. In China, this formula has historically been used to treat gastro-intestinal disorders. Previous studies have shown that YYFZBJS markedly suppresses CRC progression in ApcMin/+ mice by remodeling the gut microbiota and inhibiting the generation of regulatory T cells (4). Another study found that YYFZBJS inhibits the development of ETBF-induced colorectal tumors in mice, as well as *in vitro* CRC cells proliferation through the regulation of ETBF primed Bone marrow-derived macrophages (5). Notably, the combination of Fuzi with radiotherapy exhibits significant anti-proliferative efficacy, strengthens immune response, suppresses immune escape, and weakens immune suppression in several types of human cancer cells (6, 7). Herba Patrinia ethanol extract (PE) triggers apoptosis of CRC cells and inhibits CRC tumor angiogenesis and proliferation (8). Like TCM formula, YYFZBJS contains several components and modulates several targets and cancer signaling pathways. Currently, the bioactive ingredients and mechanisms by which it inhibits CRC are not fully understood. Here, the inhibitory effects of YYFZBJS were explored to uncover its underlying mechanisms.

Network pharmacology combines systems biology, polypharmacology, and molecular networks to comprehensively describe the relationship between drugs, their targets, and diseases in terms of a visual active ingredients–targets–diseases network (9). Molecular docking involves modeling interactions between small molecules and proteins and its use in investigating the mechanisms of action of new drugs has markedly increased in recent years. Thus, network pharmacology and molecular docking offer unique advantages and have great potential in the study of TCM.

**Figure 1:**
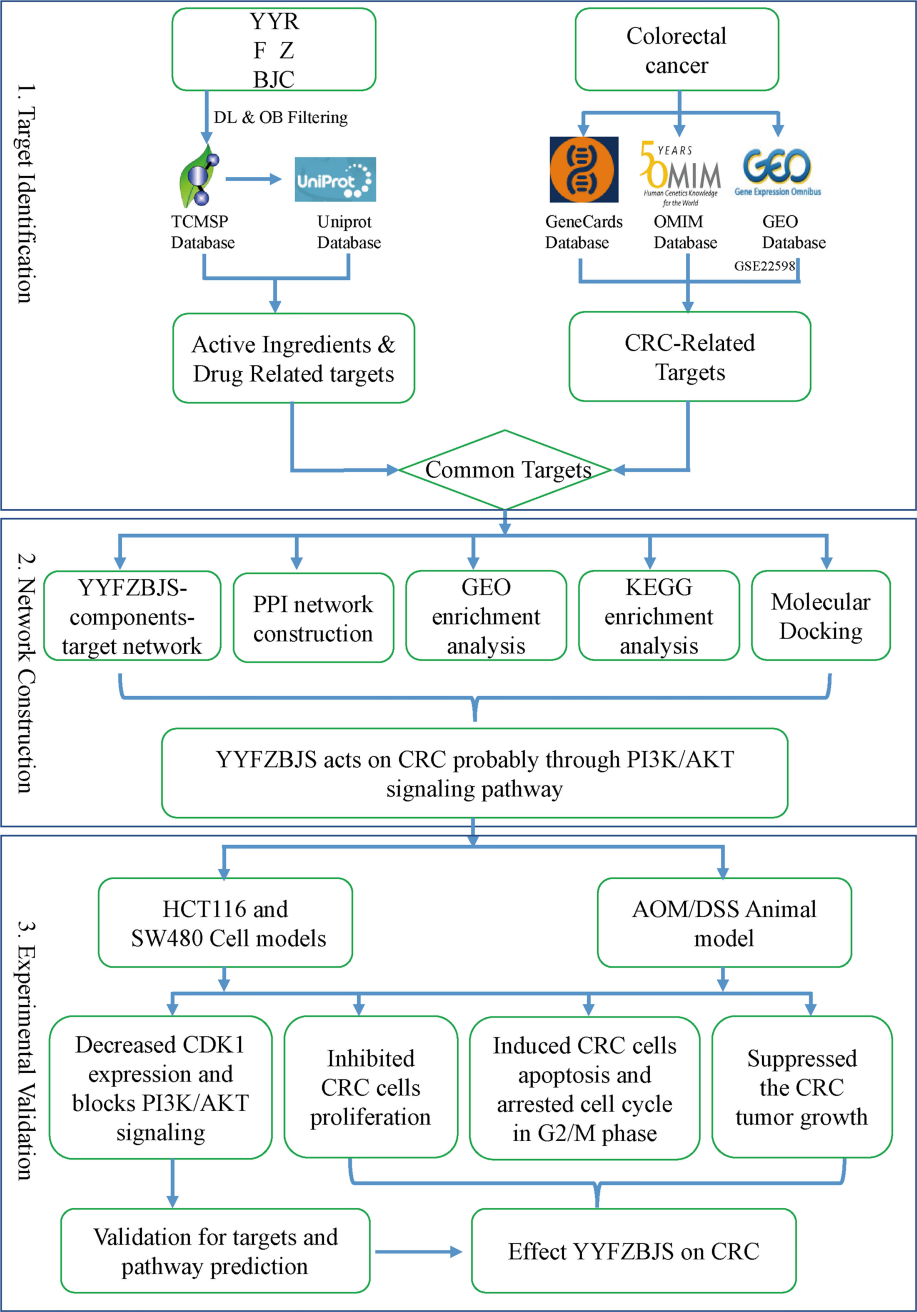
A flowchart of this study based on network pharmacology, molecular docking, and experimental validation of the mechanisms underlying the effects of YYFZBJS in colorectal cancer.

Here, the molecular mechanisms mediating the effects of YYFZBJS on CRC were elucidated (**Figure 1**).

## Material and methods

### Herbal data collection

Active YYFZBJS compounds were identified on the Traditional Chinese Medicine Systems Pharmacology (TCMSP) database (https://tcmsp-e.com) (10). In this study, oral bioavailability (OB) and drug-likeness (DL) were adopted to identify the bioactive ingredients of YYFZBJS. OB described the percentage of an oral dose capable of producing pharmacological activity. DL was applied to determine the similarity of a compound to conventional drugs and its physicochemical properties. Only compounds that had related targets and met the ADME criteria (ie, OB threshold≥30% and DL threshold≥0.18) were included in the study for subsequent research (11). Additionally, drug-related targets (DRTs) and their gene symbols were obtained from TCMSP and UniProt (https://www.uniprot.org), respectively.

### Related targets of CRC

Information on disease targets was obtained using datasets from GEO (https://www.ncbi.nlm.nih.gov/gds), Online Mendelian Inheritance in Man Database (OMIM, https://omim.org) and GeneCards (https://www.genecards.org). The term “Colorectal Cancer” was searched in OMIM and GeneCards to obtain all CRC targets, as well as in GEO to obtain dataset GSE22598 (Patient data) and GPL570 platform files. The R (version 3.6.0) package, LIMMA, was used to identify differentially expressed genes (DEGs) using p≥0.05 and log2 fold change (FC) ≥1 as cutoff thresholds. Finally, we summarized the differentially expressed genes by taking the intersection of the DEGs identified from the GSE22598 and the CRC targets acquired from GeneCards and OMIM.

### Construction of the YYFZBJS–targets–drug and protein–protein interaction networks

Using R (version 3.6.0), the drug-disease common targets were identified and visualized on a Venn diagram. Using Cytoscape (version 3.8.2), the protein–protein interaction (PPI) network between putative YYFZBJS targets was combined with that of the CRC-related targets. Next, the Cytoscape plugin, CytoNCA, was used to identify nodes with topological importance in the interaction network based on betweenness centrality (BC) and degree centrality (DC). These variables reflect the topological significance and their applications in systems pharmacology and network pharmacology have been previously described (12).

### Gene ontology and Kyoto encyclopedia of genes and genomes analyses

Gene ontology (GO) biological process enrichment analysis was conducted for the common drug targets on R’s bioconductor environment (http://www.bioconductor.org/) and used histograms and bubble charts to present the top 20 processes (p <0.05). Kyoto encyclopedia of genes and genomes (KEGG) pathway enrichment analysis was done using the R packages “clusterProfiler” and “pathview” and the top 20 KEGG pathways visualized on heatmaps. The network of functional pathways was developed using Cytoscape.

### Molecular docking

Molecular docking analysis was done using the molecular operating environment (MOE) v2015.10 in order to validate compound–target interactions. Protein 3D structures from the protein data bank (PDB, http://www.rcsb.org/) were imputed it into MOE to construct mating pockets of molecular docking after deleting water molecules, preparing protein structure, and minimizing energy. The structures of the compounds used for mating pocket construction and molecular docking analysis were downloaded from PubChem (https://pubchem.ncbi.nlm.nih.gov/) (13).

### YYFZBJS preparation

The composition of YYFZBJS was 60g Yi-yi-ren (Semen Coicis), 12g Fu-Zi (monkshood), and 30g Bai-jiang-cao (Herba Patriniae) were purchased from Union hospital of Tongji Medical College, Huazhong University of Science and Technology under the guidance of Chinese Pharmacopeia 2020 edition. The extracts of the above variously prepared YYFZBJS were obtained by the following process. Firstly, all herbs were soaked for 30 min and decocted twice independently with the 8-fold volume of water, 1 h each time. Secondly, the decoction was collected and filtrated through filter paper. Thirdly, an 8-fold volume of 95% ethanol was added to the filtrate remains and reflux twice, 1 h each time. Next, these two parts of filtrates were combined, condensed to a density of 1.0 g/mL by rotary evaporation at 55°C and frozen at -80°C. Finally, it was converted into lyophilized powder by a freeze-drying machine (Cuddon; Blenheim, New Zealand). For *in vitro* assays, the YYFZBJS lyophilized powder was dissolved in culture media by ultra-sonication for 20 min, followed by centrifugation at 13,000 rpm for 5 min. The obtained supernatant was filtered with a 0.22 μm filter membrane as the extract solution. Before using, the extract solution was diluted to the desired concentrations. Culture media without YYFZBJS was used as negative control. For *in vivo* experiments, mice were intragastrically administered a YYFZBJS decoction.

### Azoxymethane/dextran sulfate sodium induced CRC model and YYFZBJS treatment

Fifty male C57BL/6J (6–8 weeks) mice were obtained from the SPF (Beijing) biotechnology Co., Ltd. The animals were housed in pathogen-free conditions. CRC was induced using azoxymethane (AOM, Sigma Chemical Co) and dextran sulfate sodium (DSS, MP Biomedicals) as described before (14). Briefly, the mice were intraperitoneally (I.P.) injected with AOM (12 mg/kg) on day 1. After 1 week, they were administered 2.5% DSS in drinking water for 7 days and given tap water for 14 days to allow recovery. This cycle was repeated thrice and the mice sacrificed 2 weeks after the final DSS cycle. In the clinical practice of Chinese herbal medicine, YYFZBJS is usually prescribed at 51 mg daily. After converting the human dose into a mouse dose (assuming a person of 60 kg and a conversion factor of 9 between a person and a mouse), the equivalent mouse dose was 7.65 g/kg. Based on this human equivalent dose, the mice were administered YYFZBJS at 3.825 g/kg, 7.65 g/kg, and 15.3 g/kg once day from the second week for 10 weeks during the DSS treatment separately, with the control group receiving the same volume of sterile isotonic saline. The animals were provided normal drinking water. Mouse health and weight were monitored daily and weekly, respectively.

### Cell culture and transfection

The human CRC cell lines, HCT116 and SW480, were purchased from the American Type Culture Collection (ATCC, Manassas, VA, United States) and confirmed to be mycoplasma-free using a LookOut mycoplasma PCR detection kit (Sigma Chemical Co). SW480 cells were cultured in DMEM/high glucose media (Gibco, USA). HCT116 cells were cultured in McCoy’s 5A media (Gibco, USA) supplemented with 10% FBS (Gibco, USA) at 37 °C, 5% CO2.

HCT116 and SW480 cells (4×105 per well) were seeded in six-well plates and then transfected with a CDK1 overexpression plasmid (Guangzhou RiboBio Co., Ltd, Guangzhou, China) using Lipofectamine^®^2000 (Invitrogen, USA) according to manufacturer instructions. After 6 h, the transfection mixture was replaced with fresh growth media.

### Histology

Mice were sacrificed and whole intestines collected and washed with ice-cold phosphate-buffered saline (PBS). To assess inflammation, the length of the whole colon was measured before and after being opened longitudinally (15). Adenoma incidence was determined by counting the number of tumors visible in the intestine. Tissues were fixed in 4% paraformaldehyde and embedded in paraffin. Next, the sections were stained with hematoxylin and eosin (HE) and examined by a pathologist who was blinded to the experimental groups.

### Cell counting kit-8 assay

Cell proliferation was assessed using the cell counting kit-8 (CCK-8) assay (GLPBIO, Montclair, CA, USA). HCT116 and SW480 were seeded at 3 × 103 cells/well in 96 well plates and cultured to 80% confluence. They were then treated with YYFZBJS at 0, 30, 60 and 90 μg/mL for 24 h. Next, 10µL of CCK-8 solution was added into each well and the cells incubated for 90 min followed by absorbance (450 nm) reading on a microplate reader (Thermo Fisher, USA). The data are representative of three independent experiments in triplicate. IC50 values were calculated from the percent of viability reported to untreated cells using online tools https://www.xiantao.love/products.

### Colony formation assays

HCT116 and SW480 cells were treated with 30, 60 and 90 μg/mL of YYFZBJS or PBS as a control for 24 h. The cells (3 × 103 per dish) were then cultured in 10 cm dish, and the medium was changed every 5 days for 14 days. Next, the colonies were fixed with 2% paraformaldehyde (Beyotime Biotechnology, Shanghai, China) for 30min and stained with 0.1% crystal violet (Beyotime Biotechnology, Shanghai, China) for 15min. They were then washed thrice, dried, and the number of positively stained colonies counted and imaged under a microscope (Leica, Germany).

### Cell cycle analyses

HCT116 and SW480 cells were treated with YYFZBJS (30, 60 or 90 μg/mL) or PBS (negative control) for 24 h. The cells (1×106) were then collected, fixed in cold ethanol, and stored at 4°C overnight. The next day, they were washed twice with cold PBS and stained with 200μL PI/RNase staining buffer (BD Biosciences, USA) at 4 °C for 30 min in the dark. Measurements were taken using a flow cytometer (Beckman Coulter, USA) and the data analyzed using FlowJo 8.1 software.

### Apoptosis analyses

Apoptotic cells were detected using an Annexin V-APC/7-ADD apoptosis detection kit (BD Biosciences, USA). Briefly, cells were detached using EDTA-free trypsin, centrifuged, and the supernatant discarded. They were then rinsed with ice-cold PBS, centrifuged, and the cell pellet resuspended in 200 μl of 1 × binding buffer. The cells were then transferred into a FACS tube and stained with Annexin V-APC and 7-AAD (5µL each) in the dark for 15 mins, at room temperature. Next, flow cytometry measurements were taken, and the data analyzed on FlowJo 8.1.

### Western blot analysis

Total protein was extracted from the YYFZBJS-treated CRC cells or mouse CRC tissues using RIPA lysis buffer supplemented with a phosphatase and protease inhibitor cocktail. Protein concentration was then measured using a BCA assay kit according to manufacturer instructions. Next, 40μg of each sampled were resolved using 10% SDS-PAGE and transferred onto 0.45μm PVDF membranes (Millipore, USA). Membranes were then blocked using 5% skimmed milk in TBST for 1 h at room temperature, and then washed with TBST. The membranes were then incubated overnight (at 4°C) with antibodies against CDK1(1:1,000; Abcam), p-PI3K (1:1,000; CST), PI3K (1:1,000; CST), p-AKT (1:1,000; CST), AKT (1:1,000; CST) and β-actin (1:2000, ABclonal). The membranes were then washed and incubated with HRP-conjugated goat anti-mouse or anti-rabbit (1:1000) secondary antibodies for 2 h, at room temperature. Signal was then developed using an ECL kit.

### Statistical analysis

Statistical analysis was done using SPSS (version 26.0) and data plotted for visualization using GraphPad Prism (version 8). Data were expressed as mean ± SD. For normally distributed data, independent sample t test and one way ANOVA were used to statistically compare two groups and multiple groups, respectively. Non-normally distributed data were compared using nonparametric rank sum test. p<0.05 indicated statistically significant differences.

## Result

### YYFZBJS chemical compounds and targets

A total of 40 active YYFZBJS components were retrieved (oral bioavailability: ≥30%, drug likeness: ≥0.18) from TCMSP (**Supplementary Table 1**). The corresponding genes were then converted into gene symbols using Uniprot. Upon removal of duplicates and nonhuman targets, 197 drug targets were obtained.

### Identification of candidate protein targets associated with CRC

**Figure 2:**
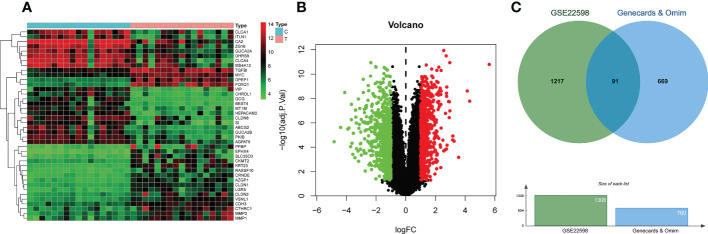
CRC-related differential gene analysis and CRC target collection. **(A)** Heatmap of differential gene analysis on dataset GSE22598. **(B)** Volcano plot of differential gene analysis on dataset GSE22598. **(C)** CRC common differentially expressed genes collection.

Analysis of screening data from OMIM and GeneCards revealed 168 and 679 CRC-related targets respectively. After merging the results from OMIM and GeneCards, and duplicate removal, 760 targets were obtained. Analysis of colorectal cancer dataset GSE22598 from GEO and its associated platform file (GPL570), identified 1308 DEGs, which were visualized on heatmaps and volcano plots (**Figures 2A, B**). As shown in **Figure 2C**, we obtained 91 differentially expressed genes by taking the intersection of the DEGs identified from the GSE22598 and the CRC targets acquired from Genecards and OMIM using an online tool (http://bioinformatics.psb.ugent.be/webtools/Venn/) (**Figure 2C**).

### Target protein–protein interaction network analysis

**Figure 3:**
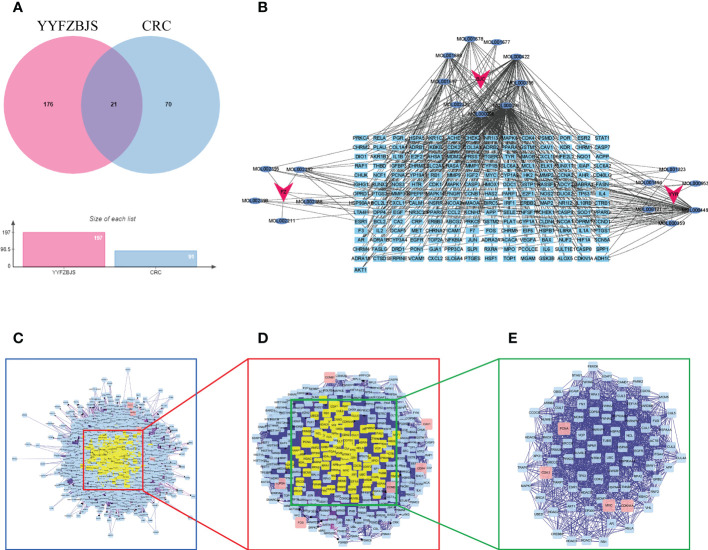
Identification of YYFZBJS’s candidate targets in CRC. **(A)** Venn diagram showed 21 common targets of YYFZBJS in CRC. **(B)** “YYFZBJS-components-target” network (red V represents herbs; blue oval represents YYFZBJS compounds; sky blue rectangle represents compound targets). **(C)** An interactive PPI network of YYFZBJS-CRC target genes. **(D)** PPI network of significant proteins extracted from **(C, E)** PPI network of candidate YYFZBJS targets for CRC treatment extracted from **(D)**. DC, degree centrality; BC, betweenness centrality; CC, closeness centrality.

A total of 197 genes related to YYFZBJS, and 91 genes related to CRC were screened out. Next, Venn diagram analysis revealed 21 common targets between the YYFZBJS related genes and the CRC related genes (**Figure 3A**). We then used Cytoscape 3.8.2 to develop a CRC-target drug network based on the 21 common targets and 91 CRC-related active components of the 3 herbs (**Figure 3B**). The active components were presented as TCMSP MolIDs using different colors to indicate which herbs they belong to. The YYFZBJS active components with the most target genes were Mol000006 (luteolin), Mol000098 (quercetin), Mol000422 (kaempferol), MOL000359 (sitosterol), and MOL000449 (stigmasterol). Thus, we speculated that these active components may contribute to the effects of YYFZBJS against CRC.

### Identification of candidate targets for YYFZBJS against CRC

To better understand the effects of YYFZBJS on CRC, we merged the PPI network of its putative targets and the PPI network of CRC-related targets in order to identify candidate YYFZBJS targets in CRC. This network contains 2,308 nodes and 64,565 edges (**Figure 3C**). Next, a subnetwork containing 283 nodes and 9,376 edges was obtained by selecting the top 30% based on degree centrality (DC, **Figure 3D**). After screening the subnetwork’s top 30% based on betweenness centrality (BC), a core network containing 85 nodes and 1516 edges was constructed (**Figure 3E**). Based on the core network, higher degrees indicated larger node sizes; magenta indicates the highest degree. The nodes with the top four degrees were CDKN1A, MYC, PCNA, and CDK1, which identified as critical genes in the PPI and subjected to further analysis.

### GO enrichment analysis of YYFZBJS-CRC common targets

**Figure 4:**
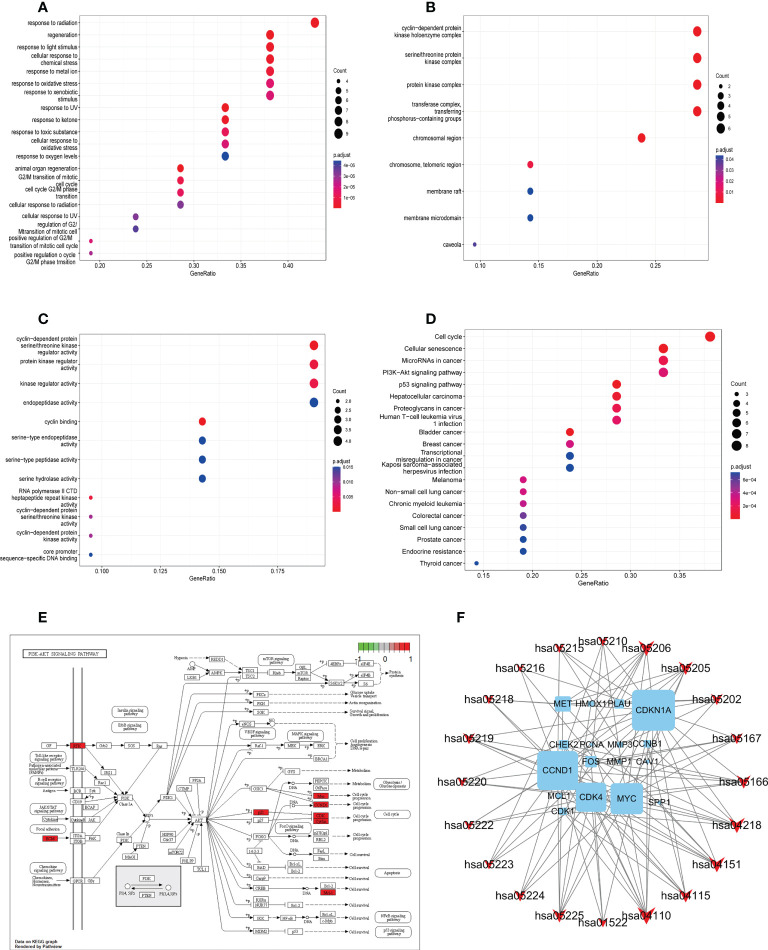
Gene ontology and KEGG pathway enrichment analysis of identified YYFZBJS-CRC common targets. **(A)** Biological processes. **(B)** Cellular components. **(C)** Molecular functions. **(D)** KEGG pathway enrichment analysis of key targets. The color scale indicates the p value, and the dot size indicates the gene count in each term. **(E)** Schematic of the PI3K/Akt signaling pathway. **(F)** Gene–pathway network of YYFZBJS against CRC. Sky blue squares represent target genes. Red V shapes represent pathways. Big size of the dot represents higher betweenness centrality.

To determine the function of the identified protein targets, we performed functional enrichment analysis of the target genes. The R package, clusterProfiler, was used to annotate the biological process (BP), molecular function (MF), and cell composition (CC) of the 21 hub target proteins. The top 20 BP, CC, and MF terms (ranked based on their adjusted p value) are shown in **Figures 4A–C**. Low adjusted p values and red color indicated greater GO term enrichment. A total of 273 GO terms, including 252, 9, and 12 terms belonging to BP, CC, and MF, respectively, were identified. Most BP GO terms were associated with response to radiation, regeneration, response to light stimulus, cellular response to chemical stress, response to metal ions, response to oxidative stress, and response to xenobiotic stimulus. The main CC terms were associated with transferring phosphorus-containing groups, transferase complex, protein kinase complex, serine–threonine protein kinase complex, and cyclin-dependent protein kinase holoenzyme complex. MF terms were mainly enriched for kinase regulator activity, cyclin-dependent protein serine–threonine kinase regulator activity, protein kinase regulator activity, and endopeptidase activity.

### Pathway enrichment and construction of the compound–target–pathway network

We used KEGG pathway analysis to identify potential pathways underlying the effects of YYFZBJS in CRC treatment. KEGG annotation revealed that 149 of the 163 target genes were enriched for 97 pathways (p<0.001). The top 20 significantly enriched pathways are shown. Furthermore, KEGG enrichment analysis showed that several target genes were closely associated with the cell cycle, cellular senescence, microRNAs in cancer, PI3K/Akt signaling, and p53 signaling pathway (**Figure 4D**). Among these pathways, PI3K/Akt signaling was the most prominent pathway based on the number of genes (**Figure 4E**), suggesting that YYFZBJS may activate P13K–Akt signaling in CRC. The PI3K/Akt pathway modulates cell proliferation, cell cycle, differentiation, apoptosis, and migration *via* multiple factors, including EGFR, BCL2, CCND1, CDK1, and CDK4. Next, a gene–pathway network was constructed using the significantly enriched pathways. Genes that influence these pathways are shown in **Figure 4F**.

### Molecular docking verification of core targets and active ingredients

**Figure 5:**
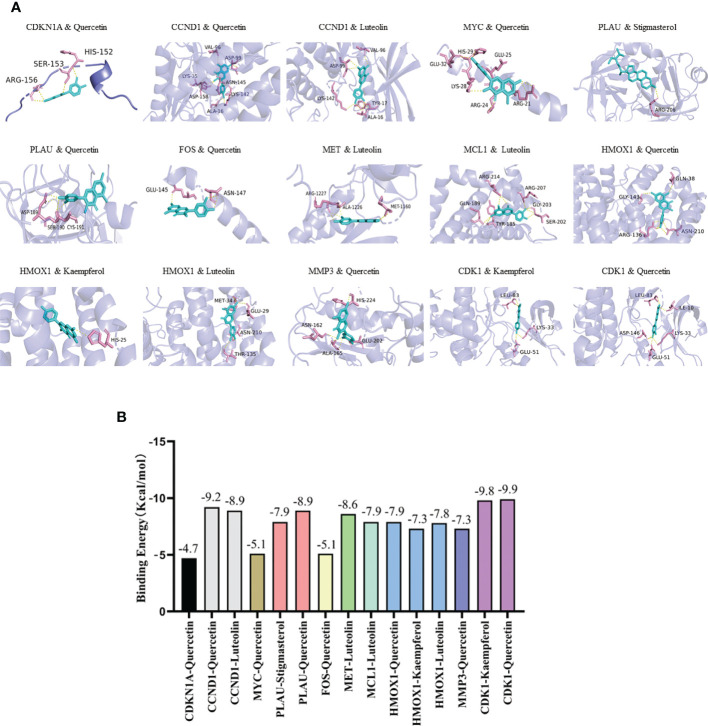
Molecular docking results. **(A)** Docking patterns of key targets genes (CDKN1A, CCND1, CDK1, MYC, PLAU, FOS, MET, MCL1, HMOX1, and MMP3) and specific active ingredients (quercetin, luteolin, kaempferol, stigmasterol, and sitosterol) of YYFZBJS. **(B)** Binding affinities (kcal/mol) of key targets and specific active ingredients of YYFZBJS.

Five active ingredients (quercetin, luteolin, kaempferol, stigmasterol, and sitosterol) and ten potential target genes (CDKN1A, CCND1, CDK1, MYC, PLAU, FOS, MET, MCL1, HMOX1, and MMP3) were selected through network analysis, with high node scores and confidence. Molecular docking studies were conducted to identify the interactions between the active YYFZBJS ingredients and CRC-related potential target genes at the molecular level. The 3D structures of the active ingredients and potential target genes were obtained from TCMSP and RCSB PDB, respectively. After dehydration and hydrogenation, we performed docking analysis using AutoDock tools and the AutoDock Vina software. The absolute binding energy value correlated positively with the connective stability of the ingredient molecule and target protein. The most stable connective patterns and binding energies are shown on **Figures 5A, B**.

According to the scoring results, quercetin-CDK1, kaempferol-CDK1, quercetin-CCND1, luteolin-CCND1, quercetin-PLAU, luteolin-MET had a strong correlation. Based on network analysis and molecular docking simulation, quercetin, which targets multiple CRC-related targets, with strong binding to the cell cycle factor, CDK1, was selected as candidate drug for *in vivo* analysis

### Clinical significance of target hub genes in CRC

**Figure 6:**
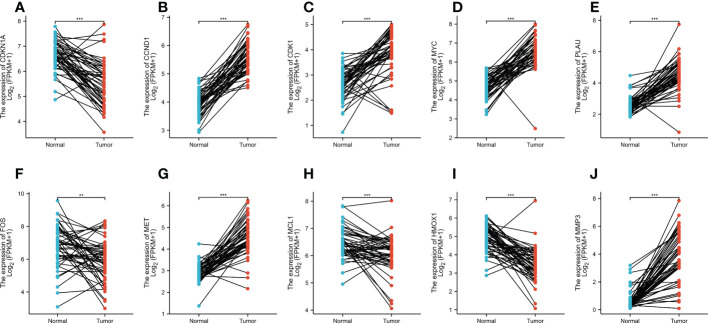
**(A–J)** The expression levels of CDKN1A, CCND1, CDK1, MYC, PLAU, FOS, MET, MCL1, HMOX1, and MMP3 in normal tissues versus CRC tissues based on the TCGA dataset. **p < 0.01, ***p < 0.001 versus the Normal.

In total, 50 tumor samples and paired adjacent noncancerous tissue from TCGA (https://tcga-data.nci.nih.gov/tcga/) were analyzed. The publicly available data indicated that among the core target proteins, the levels of CDKN1A, CCND1, CDK1, MYC, PLAU, FOS, MET, MCL1, HMOX1, and MMP3 were significantly different in CRC tissues when compared with normal colorectal tissues (**Figure 6**). The levels of CDKN1A, FOS, MCL1, and HMOX1 were high in CRC tissue and low in normal colorectal tissue, while those of CCND1, CDK1, MYC, PLAU, MET, and MMP3 were significantly higher in CRC tissues than in normal colorectal tissue.

### YYFZBJS inhibits the proliferation of human CRC cells

**Figure 7:**
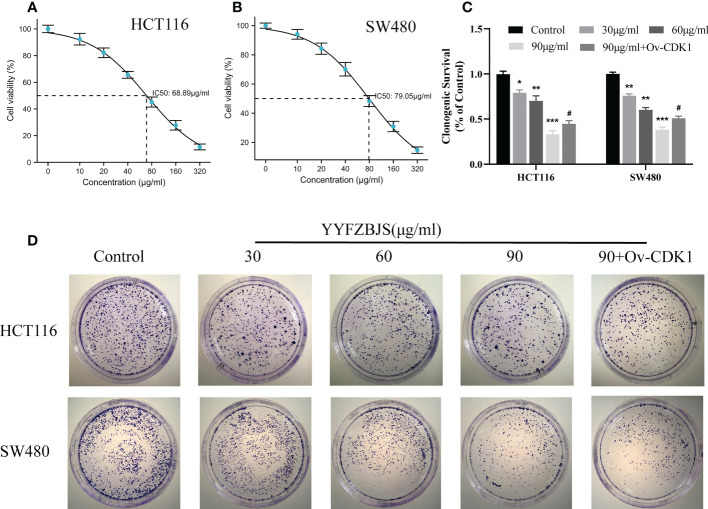
YYFZBJS inhibits cell viability. Viability of **(A)** HCT116 and **(B)** SW480 cells after treatment for 24 h with indicated doses of YYFZBJS. All data are presented as mean  ± SD (n=3). **(C)** Colony formation assays were done using HCT116 and SW480 cells treated with YYFZBJS. A representative of three experiments is shown. **(D)** Colony formation is presented as mean ± SD (n=3), *p < 0.05, **p < 0.01, ***p < 0.001 versus the control group. ^#^p < 0.05 versus the YYFZBJS 90 μg/ml group.

To assess its effects on human CRC cells, cultured HCT116 and SW480 cells were treated with YYFZBJS at 0, 30, 60, and 90 μg/mL for 24 h and cell viability examined using CCK8 analysis. This analysis revealed that YYFZBJS significantly suppressed the viability of HCT116 and SW480 cells (**Figures 7A, B**). Colony formation assay, revealed that YYFZBJS suppressed colony formation by HCT116 and SW480 cells at all indicated concentrations (**Figures 7C, D**). Five active compounds including quercetin, kaempferol, beta-sitosterol, isorhamnetin, and stigmasterol were observed to inhibit SW480 and HCT116 proliferation in a concentration-dependent manner. The 24 h IC50 values of quercetin, luteolin, kaempferol, stigmasterol, and sitosterol in SW480 were 25.63, 85.89, 43.54,58.97, and 83.13μM; meantime the 24 h IC50 values of quercetin, kaempferol, beta-sitosterol, isorhamnetin, and stigmasterol in HCT116 were 27.58, 99.56, 41.28, 64.49, and 67.79μM. The cell viability curves were shown in **Supplement Figure 1**. These data indicate that YYFZBJS inhibits CRC cell viability and proliferation.

### YYFZBJS induces apoptosis and arrests cell cycle progression in the G2/M phase by inhibiting CDK1/PI3K/AKT signaling

**Figure 8:**
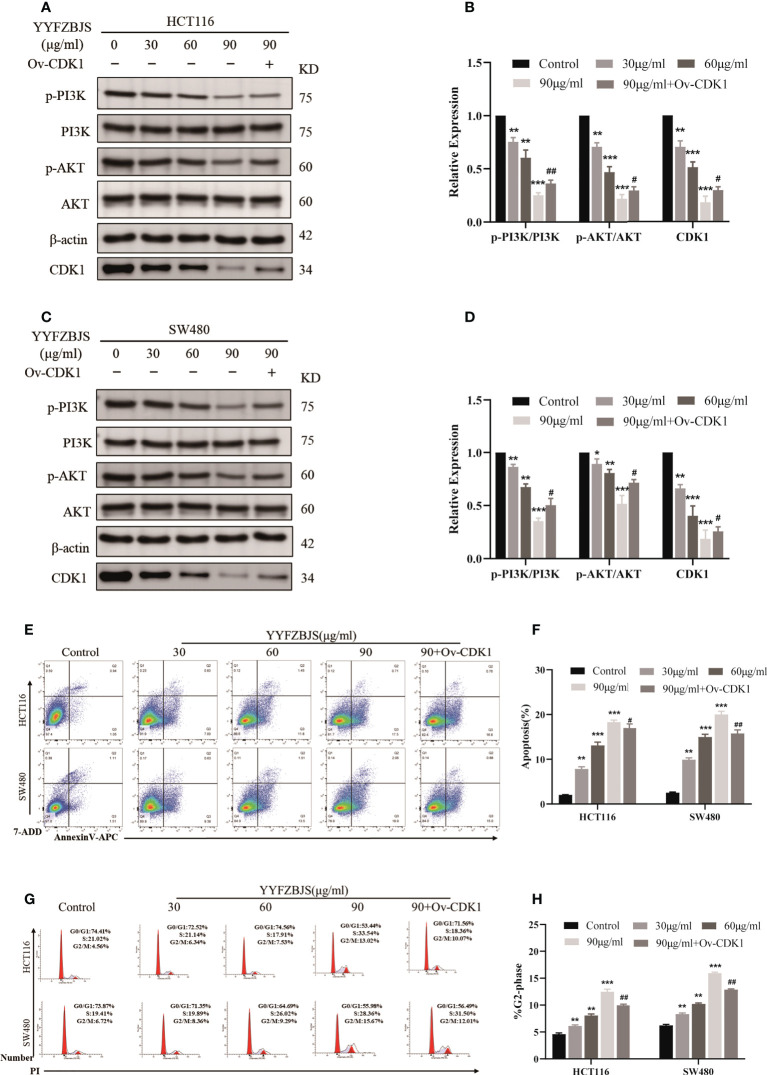
In HCT116 and SW480 cells, YYFZBJS induces apoptosis and cell cycle arrest in the G2/M phase, and inhibits CDK1/PI3K/AKT signaling. **(A–D)** Western blot analysis revealed that YYFZBJS (30, 60, and 90 µg/mL) dose dependently suppresses CDK1 expression and PI3K/AKT signaling in HCT116 and SW480 cells. Transfecting the cells with overexpressed CDK1 plasmid reversed YYFZBJS-induced PI3K/AKT expression in HCT116 and SW480 cell lines. **(E)** Flow cytometric analysis of Annexin V-APC/7-ADD double staining in HCT116 and SW480 cells treated with various concentrations of YYFZBJS for 24 h. **(F)** Statistical analysis of the apoptotic rate in HCT116 and SW480 cells. **(G)** Flow cytometric analysis of cell cycle arrest in HCT116 and SW480 cells treated with indicated doses of YYFZBJS for 24 h. **(H)** Quantification of cells in the G2/M phase of the cell cycle. All data are presented as mean ± SD (n=3). *p < 0.05, **p < 0.01, ***p < 0.001 versus the control group; ^#^p < 0.05, ^##^p < 0.01 versus the YYFZBJS 90 μg/ml group.

Next, we treated the CRC cells, HCT116 and SW480, with YYFZBJS at 0, 30, 60, and 90 μg/mL for 24 h and assessed the levels of CDK1 and PI3K/AKT using western blot analysis. This analysis revealed that YYFZBJS dose-dependently inhibited CDK1, p-PI3K and p-AKT expression in HCT116 and SW480 cells, which was reversed by CDK1 overexpression (**Figures 8A–D**). These results indicated that YYFZBJS could not only alter the expression of CDK1 and PI3K/AKT in colon cancer cells, but that it also has a regulatory relationship with CDK1. In addition, we performed different concentrations of YYFZBJC intervention in HCT116 and SW480, while overexpressing CDK1 in the 90μg/mL group. Then, the cell apoptosis and cell cycle distribution were investigated through flow cytometry. The results showed that administration of YYFZBJS at 30, 60, and 90μg/mL significantly increased the rate of apoptosis (**Figures 8E, F**). Moreover, YYFZBJS induced cell cycle arrest in HCT116 and SW480 cells (**Figures 8G, H**). Upon CDK1 overexpression, YYFZBJS’s ability to induce apoptosis and cell cycle arrest in the tumor cells was weakened (**Figures 8E–H**). These results suggest that YYFZBJS induced apoptosis and cell cycle arrest in the G2/M phase by inhibiting CDK/PI3K/AKT signaling.

### YYFZBJS inhibited tumor growth and PI3K/AKT signaling in a mouse model of CRC

**Figure 9:**
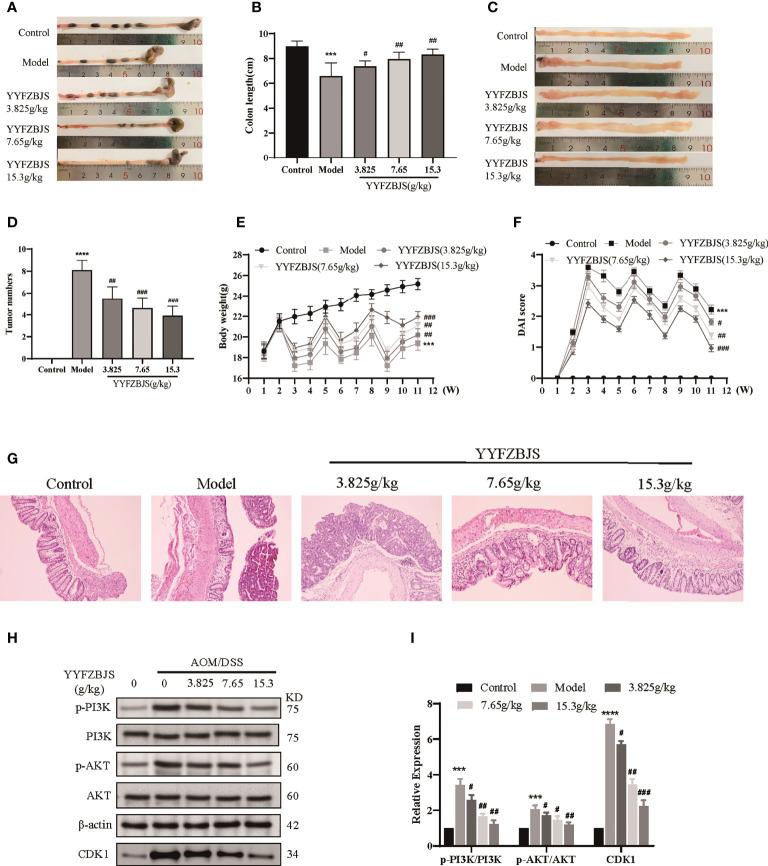
YYFZBJS inhibits colorectal cancer progression in mice through suppression of the CDK1/PI3K/Akt pathway. **(A, B)** YYFZBJS suppresses intestinal shortening in the mouse model of colon cancer (n=10). **(C, D)** YYFZBJS inhibits tumorigenesis in the mouse model of colon cancer (n=10). **(E)** YYFZBJS slowed weight loss in the mouse model of colon cancer (n=10). **(F)** YYFZBJS improved intestinal DAI score in the mouse model of colon cancer (n=10). **(G)** Hematoxylin and eosin staining (H&E; magnification: 100×) analysis confirmed that YYFZBJS improved intestinal morphology after disruption by AOM and DSS and inhibited tumorigenesis. **(H, I)** Western blot analysis confirmed that the effects of YYFZBJS on the mouse model of colon cancer was mediated *via* CDK1/PI3K/Akt signaling. All data are presented as mean ± SD (n=3), ***p < 0.001, ****p < 0.0001 versus the control group; ^#^p < 0.05, ^##^p < 0.01, ^###^p < 0.001 versus the model group.

To investigate the antitumor effects of YYFZBJS, we established a mouse model of CRC using AOM/DSS and treated the mice with YYFZBJS at 3.825 g/kg, 7.65 g/kg, and 15.3 g/kg using oral gavage. The mice were then sacrificed after 11 weeks and the entire colorectal tissue (from cecum to anus) collected. We then measured colon length and counted the number of tumors. Colon length shortening is regarded as a hallmark of inflammation upon DSS treatment (16). Here, we found that the mean colon length was significantly shorter in mice treated with AOM/DSS when compared to control mice (p<0.01), Notably, YYFZBJS markedly reversed the colon length changes triggered by AOM/DSS (**Figures 9A, B**). Tumor number and size is an indicator of the modeling efficiency and treatment efficacy. Here, we found that YYFZBJS significantly reduced the number of tumors dose-dependently (**Figures 9C, D**). In the model group, we found that mice that freely drank the DSS solution for seven consecutive days in the first, second, and third cycles, exhibited marked weight loss when compared with the controls. In contrast, significant improvement in weight loss was observed in mice treated with YYFZBJS after exposure to AOM/DSS (**Figure 9E**). Compared with the control group, Disease Activity Index (DAI) score was significantly decreased in the YYFZBJS treatment group (**Figure 9F**). Notably, the number of different tumor sizes was much less in all three different doses of YYFZBJS groups than that of the model group (**Supplementary Figure 9J**). These results suggest that YYFZBJS can effectively protect against AOM/DSS-induced tumorigenesis.

HE analysis of the colon tissues revealed that the model group lost normal tissue morphology, with intestinal villi exhibiting disruption, and tissue showing apparent nuclear enlargement, aggregation, and shrinkage. Treatment with YYFZBJS partially restored normal intestinal structure and improved the above pathological changes (**Figure 9G**). Network pharmacology analysis suggested that YYFZBJS may exert its effects on CRC *via* CDK1 and PI3K/AKT signaling. Thus, we examined protein levels of CDK1, PI3K, AKT, and p-AKT in colorectal tissues using western blotting. This analysis revealed that the expression of CDK1 was reduced upon treatment with YYFZBJS at 3.825, 7.65, and 15.3 mg/kg (**Figure 8H**). Next, western blot analysis of PI3K, p-PI3K, AKT, and p-AKT levels found that YYFZBJS significantly reduced the levels of p-PI3K and p-AKT without altering total PI3K and AKT levels (**Figures 9H, I**). These results are consistent with findings from network pharmacology analyses. Taken together, these results indicate that YYFZBJS exerts its effects on CRC *via* CDK1 and PI3K/AKT signaling.

## Discussion

Using network pharmacology-based approach, experimental validation, and molecular docking tests, we explored bioactive components and the molecular mechanism of YYFZBJS for treating CRC. A YYFZBJS compound–target network constructed using 40 compounds and 197 compound targets suggested that most YYFZBJS compounds affect multiple targets. For example, luteolin, quercetin, and kaempferol affected 54, 140, and 55 targets, respectively, suggesting that they pleiotropically mediate the effects of YYFZBJS. Although each herb showed distinct numbers of putative targets, there were many overlapping targets, indicating that multiple YYFZBJS compounds may modulate the same target, resulting in synergistic effects. Anticancer agents like luteolin induce apoptosis and arrest the cell cycle, thereby inhibiting metastasis and angiogenesis in multiple cancers (17). Quercetin inhibits tumors by regulating MAPK–ERK1/2, Wnt–β-catenin, and PI3K/Akt/mTOR pathways. Quercetin also affects cancer metabolism by inhibiting key glycolysis and glucose uptake enzymes (18). Kaempferol can restore cellular redox hemostasis by inhibiting the NF-κB pathway and upregulate the Nrf2 transcriptional pathway to control cancer through its antioxidative/antinitrosative and anti-inflammatory potential (19). Our findings indicate that YYFZBJS has multitarget therapeutic effects. The role of these active compounds in CRC warrant further investigation.

We selected 40 bioactive compounds and 197 protein targets from public databases. PPI network analysis indicated that CDKN1A, CCND1, CDK1, MYC, PLAU, FOS, MET, MCL1, HMOX1, and MMP3 may be key mediators of the effects of YYFZBJS against CRC. CDK1 is a Ser/Thr protein kinase. Data indicates that CDK1–CCNB1 signaling regulates mitosis (20). CCNB1 is the main CDK1 activator and together with CDK1, promotes G2/M transition (20). Deregulated cell cycle results in formation of cancer stem cells, which drive tumorigenesis (21). CDK1 is upregulated in CRC (22). GO enrichment analysis revealed that YYFZBJS is associated with major biological processes (e.g., cyclin binding, regulator activity, protein kinase regulator, and cyclin-dependent protein serine/threonine kinase). KEGG pathway analysis revealed that multiple signaling pathways are involved in the treatment effects, including the cell cycle, cellular senescence, microRNAs in cancer, PI3K/Akt pathway, and p53 signaling. The PI3K/Akt pathway promotes cancer by influencing multiple biological processes, including cell survival, metabolism, proliferation, and cell growth (23). KEGG pathway analysis revealed that YYFZBJS’s mechanisms of action in CRC involve the cell cycle and PI3K/AKT signaling. Together, these data suggest that YYFZBJS exerts anti-CRC effects through these signaling pathways, target genes, and active compounds.

The mechanisms through which YYFZBJS act against CRC were further investigated through molecular docking analysis. Molecular docking can efficiently predict the binding affinity between TCM components and their targets based on the spatial structure of ligands and receptors. Of the ten targets chosen for molecular docking analysis, CDKN1A, CDK1, MCL1, and MYC are known to be key factors in the cell cycle and PI3K/AKT signaling. Molecular docking analysis showed that quercetin, luteolin, kaempferol, stigmasterol, and sitosterol, can interact with CDKN1A, CDK1, MYC, PLAU and MCL1, etc. Previous studies have reported that quercetin-induced growth inhibition and cell death in prostatic carcinoma cells are associated with increased CDKN1A levels (24). Quercetin inhibits proliferation in endometriosis by regulating CCND1 (25). P21 (encoded by CDKN1A), is a cyclin-dependent kinase inhibitor that prevents the phosphorylation of key cyclin-dependent kinase substrates, thereby inhibiting cell cycle progression. MYC is a key proto-oncogene but currently, there are no therapies that directly target MYC (26).

Our data show that in the CRC cells, HCT116 and SW480, YYFZBJS inhibited CDK1 expression, induced cell cycle arrest at the G2/M phase, and activated apoptosis. Moreover, upregulation of phospho-Akt (p-Akt) and PI3K in the control group indicated PI3K/Akt signaling activation. Compared with the control group, p-Akt and PI3K were significantly suppressed in the YYFZBJS group while total Akt levels were unaffected, and these effects were YYFZBJS dose-dependent. These findings indicate that YYFZBJS’s anti-CRC effects are mediated by PI3K/Akt signaling. The PI3K/Akt signaling pathway, which is dysregulated in many cancers, including CRC, may influence CRC invasion, migration, proliferation, and autophagy (27). It is also reported that CDK1/PI3K/AKT inhibitors may be effective adjuvants in CRC treatment (28).

There are several limitations to our study. Because the public databases utilized in this study are continuously updated, some bioactive ingredients and target genes may not have been included in our analysis. Additionally, other signaling pathways (e.g., P53 and cellular senescence) may be involved in the anti-tumor effects of YYFZBJS. Further research is needed to examine the potential involvement of such pathways. Moreover, YYFZBJS contains active components such as quercetin, luteolin, kaempferol, stigmasterol, and sitosterol by network pharmacology, however these are also widely distributed in many herbs/plants/fruits/vegetables and are not very specific. The aim of this paper is to investigate the effect of compound prescription on CRC. Later, we will find the unique active components of single herb and YYFZBJS in combination with high performance liquid chromatography, and further study its mechanism of action. Nonetheless, our findings provide important preliminary information on the role of YYFZBJS in CRC treatment and indicate that YYFZBJS may be a promising CRC therapeutic candidate.

## Conclusion

Our results indicate that in CRC, YYFZBJS inhibited proliferation and induced apoptosis by modulating the CDK1/PI3K/Akt signaling pathway and highlight YYFZBJS as a promising adjuvant in CRC treatment.

## Data availability statement

The datasets presented in this study can be found in online repositories. The names of the repository/repositories and accession number(s) can be found in the article/**Supplementary Material**.

## Ethics statement

The animal study was reviewed and approved by Tongji Medical College, Huazhong University of Science and Technology.

## Author contributions

SY and JL contributed to design of the study. JL, NL and FZ carried out the network pharmacology analyses. JL, LS and YL performed the experiments. JL and FZ wrote and the manuscript. LS, NL, MZ and SW contributed to manuscript revision. All authors contributed to the article and approved the submitted version.

## Funding

This work was supported by the Natural Science Foundation of China (No: 81874397), and the Project of Key R&D Program of Hubei Province (No: 2020BCA065).

## Acknowledgments

We thank all the individuals who contributed to study. We also would like to acknowledge the reviewers for their valuable comments and suggestions.

## Conflict of interest

The authors declare that the research was conducted in the absence of any commercial or financial relationships that could be construed as a potential conflict of interest.

## Publisher’s note

All claims expressed in this article are solely those of the authors and do not necessarily represent those of their affiliated organizations, or those of the publisher, the editors and the reviewers. Any product that may be evaluated in this article, or claim that may be made by its manufacturer, is not guaranteed or endorsed by the publisher.
