# Supplementary material for: Integrated network pharmacology and experimental verification to investigate the mechanisms of YYFZBJS against colorectal cancer via CDK1/PI3K/Akt signaling
Source: Front Oncol. 2022 Nov 15;12:961653. doi: 10.3389/fonc.2022.961653 (PMC9706206; doi:10.3389/fonc.2022.961653)
Supplement: Supplementary file 2 [file DataSheet_1.docx]

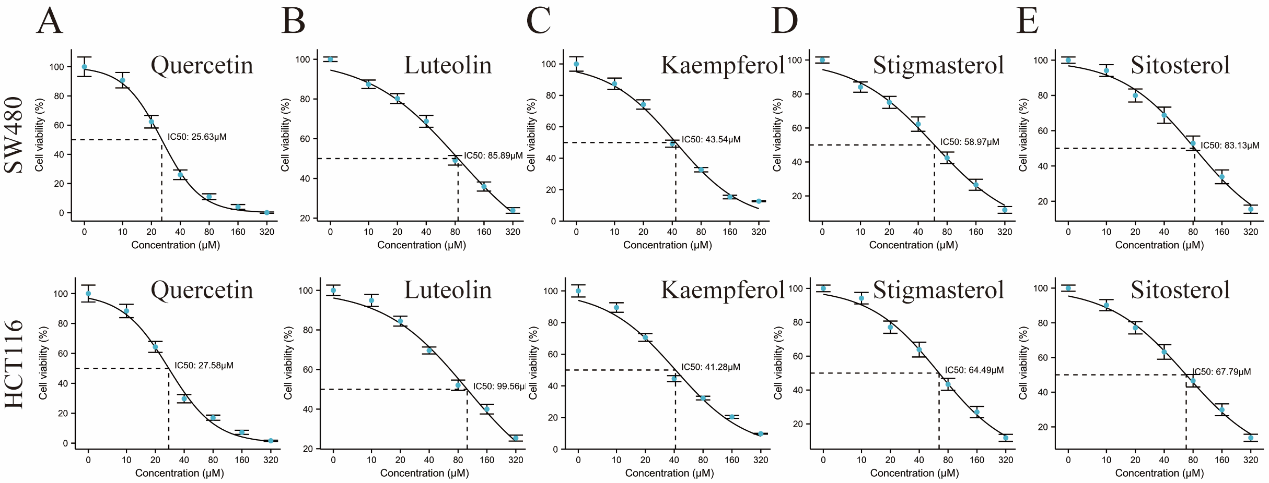


Supplement Figure 1

Proliferative inhibitory effects of five active compounds of YYFZBJS treatment, including (A)quercetin, (B)luteolin, (C)kaempferol, (D)stigmasterol, and (E) sitosterol on SW480 and HCT116 cells. Drug concentration-cell viability curves were generated based on the cell viability assay. All data were expressed as mean ± SD（n=5）
