# Supplementary figures and images for: Integrated network pharmacology and experimental verification to investigate the mechanisms of YYFZBJS against colorectal cancer via CDK1/PI3K/Akt signaling
Source: Front Oncol. 2022 Nov 15;12:961653. doi: 10.3389/fonc.2022.961653 (PMC9706206; doi:10.3389/fonc.2022.961653)

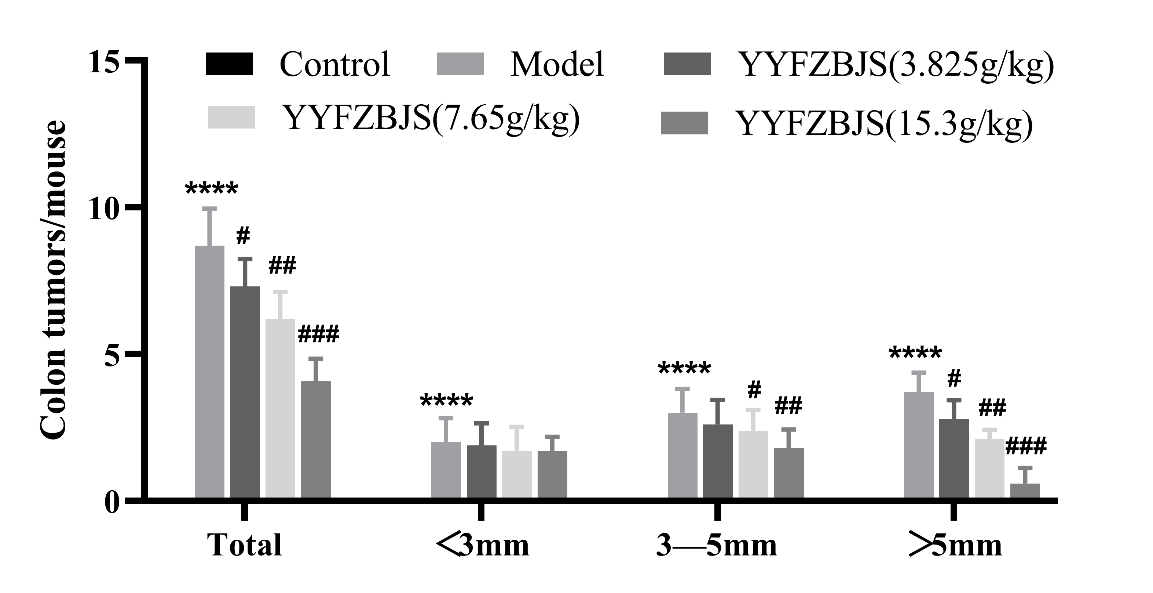


**J**

Figure9J Tumor size distribution in the colon

Supplement: Supplementary file 3 [file DataSheet_2.docx]
